# Modes of failure of Trifecta aortic valve prosthesis

**DOI:** 10.1093/icvts/ivac086

**Published:** 2022-03-28

**Authors:** Pietro Giorgio Malvindi, Hassan Kattach, Suvitesh Luthra, Sunil Ohri

**Affiliations:** 1 Wessex Cardiothoracic Centre, Department of Cardiac Surgery, University Hospital Southampton, Southampton, UK; 2 University of Southampton, Southampton, UK

**Keywords:** Aortic valve, Aortic valve replacement, Aortic valve prosthesis

## Abstract

**OBJECTIVES:**

Several concerns have been recently raised regarding the durability of Trifecta prostheses. Different mechanisms of early failure were reported. Our aim was to study in a large population the modes of failure of Trifecta valves.

**METHODS:**

We conducted a retrospective analysis of patients who underwent surgical aortic valve replacement with a Trifecta prosthesis during the period 2010–2018. Details regarding the mode of failure and haemodynamic dysfunction were collected for patients who underwent reintervention for structural valve failure. The Kaplan–Meier method was used to calculate survival. Competing risk analysis was performed to calculate the cumulative risk of reintervention for structural valve failure.

**RESULTS:**

The overall population comprises 1228 patients (1084 TF model and 144 TFGT model). Forty-four patients—mean patients’ age at the time of the first implant 69 (standard deviation: 12) years and 61% female—underwent reintervention for structural valve failure after a median time of 63 [44–74] months. The cumulative incidence of reintervention for structural valve failure was 0.16% (SE 0.11%), 1.77% (SE 0.38%) and 5.11% (SE 0.98%) at 1, 5 and 9 years, respectively. In 24/44 patients (55%), a leaflet tear with dehiscence at the commissure level was found intraoperatively or described by imaging assessment. The cumulative incidence of reintervention for failure due to leaflet(s) tear was 0.16% (SE 0.11%), 1.08% (SE 0.29%) and 3.03% (SE 0.88%) at 1, 5 and 9 years, respectively.

**CONCLUSIONS:**

Leaflet(s) tear with dehiscence along the stent post was the main mode of early failure, up to 5 years, after Trifecta valves’ implantation.

**Figure:**
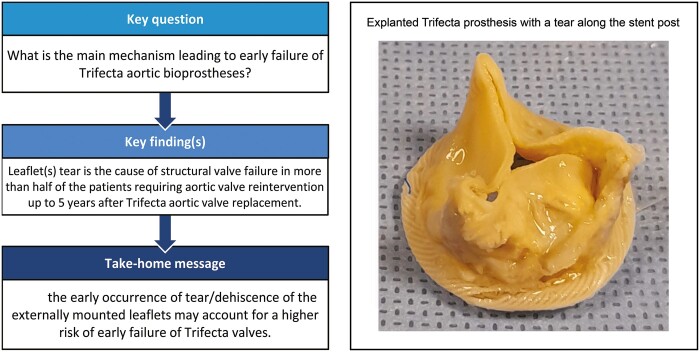

## INTRODUCTION

Trifecta (Abbott, IL, USA) is an externally mounted bovine pericardial aortic valve prosthesis. This tissue valve showed adequate haemodynamic and better early performance when compared with internally wrapped bovine pericardial prostheses [1–4]. However, several concerns have been raised regarding its durability. Recently, we have reported an increased incidence of early structural valve failure after Trifecta implantation, which was significantly higher when compared to other commonly used tissue prostheses [5]. Similarly, other experiences found a significantly increased risk of valve failure at 5–7 years since the implantation of Trifecta valves [2, 6, 7].

Different pathologic mechanisms have been associated with early failure of Trifecta valves. Alongside a progressive calcification of the cusps, several reports highlighted the development of a fibro-fatty pannus on the aortic side of the leaflets and the sudden onset of aortic regurgitation due to cusp(s) tear [8–10]. This evidence comes from limited size cohorts or case reports. The aim of our study is to describe and discuss the modes of failure of Trifecta valves in a larger population of patients.

## METHODS

### Ethical statement

Approval was obtained for the use of data (Safeguard System approval number SEV/0029, date 24.10.2018). Considering the type of the study involving anonymised and previously collected data, patients’ consent was waived.

### Population

The internal database of Wessex Cardiothoracic Centre at UHS was searched to identify patients who underwent aortic valve replacement with a Trifecta or Trifecta GT (Abbott, Abbott Park, IL, USA) tissue valve at Southampton General Hospital and Spire Hospital Southampton, during the period January 2011–February 2018.

### Study design, data collection, statistical analysis and outcome

This study is a retrospective evaluation from institutional records with prospective data entry collected and used in compliance with institutional data protection and confidentiality policies. The data were collected from the hospital database system and patients’ records.

The following data were collected: year of implant and size of the prosthesis implanted; interval time between implant and reintervention; age, haemodynamic data, morphologic details of the failed prostheses at the reintervention and patients’ survival. Continuous variables were presented as mean [standard deviation (SD)] or median (1° interquartile range, 3° interquartile range). Categorical variables were presented as numbers (%).

Structural valve failure was defined according to the standardized definitions in assessing the durability of transcatheter and surgical heart valve prostheses [11].

Survival probabilities were calculated using Kaplan–Meier curves. The occurrence of reintervention for structural valve failure was studied using cumulative incidence function with death as a competing risk. The analysis was generated using Statistical Analysis Software (SAS), Version 3.8, SAS University Edition (SAS Institute Inc., Cary, NC, USA).

## RESULTS

**Figure 1:**
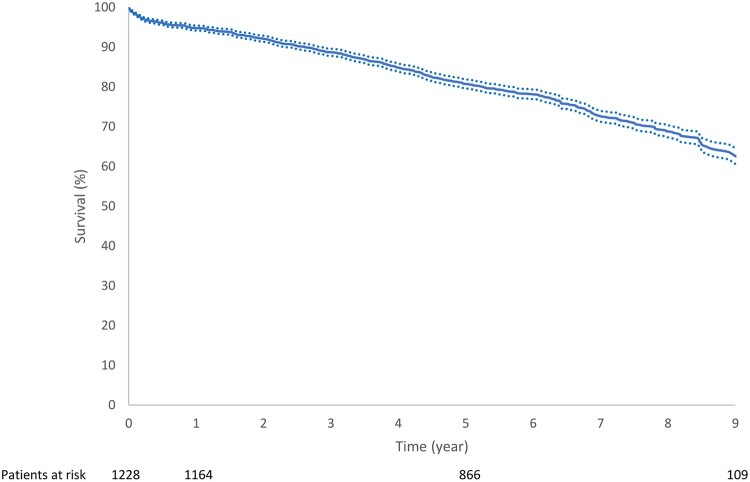
Kaplan–Meier curve of survival probabilities (± SE) of the overall population after aortic valve replacement with a Trifecta prosthesis.

A total of 1228 patients—mean age 76 (SD: 9) years—received a Trifecta prosthesis (1084 patients TF model, 144 patients TFGT model from February 2016) during the observational period. For the overall population, survival probabilities were 98.5% (SE 0.4%), 94.8% (SE 0.6%), 80.8% (SE 1.1%) and 62.5% (SE 2.0%) at 30 days, 1 year, 5 years and 10 years, respectively (Fig. 1).

**Figure 2:**
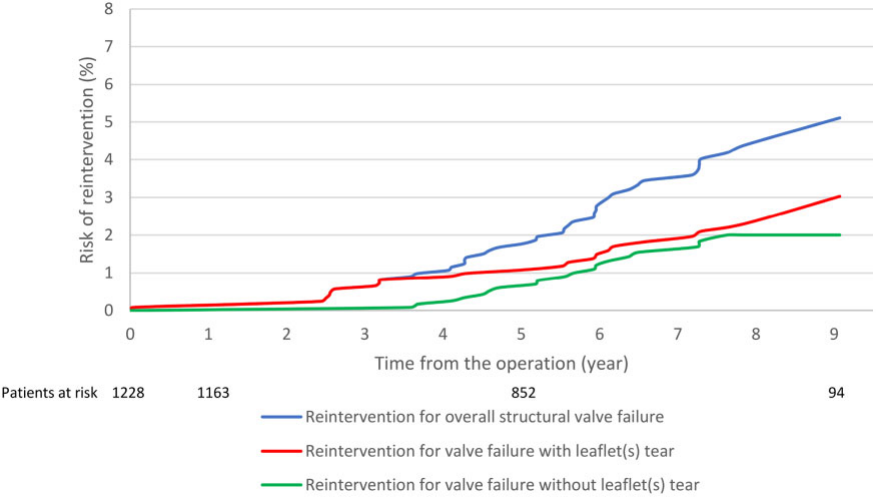
Cumulative risk probabilities of reintervention for overall structural valve failure, failure with and without leaflet(s) tear, using death as competing risk.

Forty-four patients (43 patients TF model, 1 patient TFGT model)—mean patients’ age at the time of the first implant was 69 (SD: 12) years and among them 61% (27/44) female—underwent aortic valve reintervention (surgical redo aortic valve replacement, 28 patients, or transcatheter aortic valve implantation valve-in-valve, 16 patients) for structural valve failure at a median interval time of 63 [44–74] months. The cumulative incidence of reintervention for structural valve failure was 0.16% (SE 0.11%), 1.77% (SE 0.38%) and 5.11% (SE 0.98%) at 1, 5 and 9 years, respectively (Fig. 2).

The mean patient’s age at the time of reintervention was 73 (SD: 12) years. The mortality before hospital discharge was 2.2% (1/44 patients). Aortic valve stenosis was the main indication for a reintervention in 11 patients after a median interval of 67 [56–75] months. Pure aortic regurgitation was described in 19 patients after a median interval of 38 [31–67] months. The remaining 14 patients presented mixed haemodynamic dysfunction with more than moderate aortic valve stenosis and moderate or severe aortic insufficiency; in these cases, the median interval time between valve replacement and reintervention was 73 [61–79] months.

Leaflet(s) tear with dehiscence at the commissure level was found in 24 patients. Diffuse calcification of the prosthesis leaflets with no leaflet(s) tear was the main mechanism of structural valve deterioration in 19 patients; presence of pannus on the inflow side was described in 1 case. Patients with valve calcification were reoperated after a median time of 68 [55–77] months. In patients with leaflet tear, the interval time between aortic valve implant and reintervention was 50 [31–73] months. Details of haemodynamic data, imaging or surgical findings and type of reintervention performed are reported in Table 1.

**Table 1:** Details of the 44 patients who underwent reintervention for Trifecta structural valve failure

ID	Age/gender	Year of implant	Valve size	Durability (months)	Haemodynamic abnormality	Transvalvular mean gradient (mmHg)	Peak velocity (m/s)	AR grade	LVEF (%)	Morphologic findings	Re-intervention	Survival
1	70/F	2011	21	63	Mixed	59	5.0	Moderate	53	Leaflets calcification Pannus	SAVR 21 mm (Magna Ease) + CABG + aortic root enlargement	Alive at 50 months
2	66/F	2011	23	78	Mixed	60	5.0	Moderate	60	Leaflets calcification	SAVR 23 mm (Perimount Magna Ease)	Alive at 35 months
3	81/F	2011	19	68	Mixed	23	3.1	Severe	57	Leaflets calcifications	SAVR 19 mm (Perimount Magna Ease) + MV annuloplasty	Alive at 43 months
4	63/M	2011	23	52	Regurgitation	22	3.4	Severe	52	Tear of the NCC	SAVR 23 mm (Perimount Magna Ease)	Alive at 58 months
5	27/F	2011	23	62	Stenosis	74	5.2	Mild	62	Leaflets calcification	SAVR 21 mm (On-X)	Alive at 52 months
6	76/F	2011	19	109	Regurgitation	28	3.6	Severe	55	Tear of the RCC	SAVR 19 mm (Perimount Magna Ease) + CABG	Alive at 3 months
7	86/F	2011	23	94	Mixed	51	4.8	Severe	55	Tear of NCC	TAVI 23 mm (S3)	Alive at 24 months
8	78/F	2012	23	44	Mixed	52		Moderate	57	Leaflets calcifications	SAVR 21 mm (Perimount) + MV annuloplasty + CABG	Died before hospital discharge
9	87/M	2012	23	31	Regurgitation	22	2.8	Severe	62	Tear of the RCC	SAVR 23 mm (Perimount)	Alive at 73 months
10	75/F	2012	23	72	Stenosis	51	4.4	Mild	40	Leaflets calcification	TAVI 23 mm (S3)	Alive at 32 months
11	68/M	2012	23	29	Regurgitation	26	3.7	Severe	60	Tear of the RCC	SAVR 23 mm (Perimount)	Alive at 71 months
12	86/F	2012	21	86	Stenosis			Severe	60	Leaflets calcification	TAVI 23 mm (S3)	Alive at 23 months
13	80/F	2012	21	87	Mixed	31	3.9	Severe	70	Leaflets calcification	TAVI 23 mm (S3)	Alive at 11 months
14	67/F	2012	21	55	Stenosis	64	4.0	Moderate	60	Leaflets calcification	SAVR 21 mm (St Jude mechanical)	Alive at 52 months
15	63/F	2012	23	73	Mixed	55	3.7	Severe	60	Tear of the RCC	SAVR 21 mm (Perimount)	Alive at 34 months
16	83/F	2012	21	77	Stenosis	66	4.0	Mild	60	Leaflets calcification	SAVR 19 mm (Perimount Magna Ease)	Alive at 42 months
17	66/F	2012	21	79	Mixed	36	4.1	Severe	60	Tear of the RCC and NCC	SAVR 21 mm (Perimount Magna Ease)	Alive at 26 months
18	55/F	2012	23	51	Stenosis	38	4.1	Trivial	60	Pannus formation	SAVR 23 mm (Perimount Magna Ease)	Alive at 52 months
19	66/M	2012	23	71	Stenosis	62	4.84	None	60	Leaflets calcification	SAVR 23 mm (Perimount Magna Ease) +Aortic root enlargement	Alive at 31 months
20	82/F	2012	21	51	Mixed	38	4.0	Moderate	60	Leaflets calcification	SAVR 19 mm (Perimount) +Aortic root enlargement	Alive at 49 months
21	69/M	2012	25	92	Stenosis	64	4.9	Mild	50	Leaflets calcification and pannus formation	SAVR 25 mm (Perimount)	Alive at 9 months
22	91/M	2012	21	88	Regurgitation			Severe	45	Leaflet tear	TAVI 23 mm (S3)	Died after 14 months
23	80/M	2012	23	38	Regurgitation			Severe	65	Tear of the NCC	SAVR 23 mm (Perimount)	Alive at 70 months
24	70/F	2013	19	31	Regurgitation	17		Severe	52	Tear of the NCC	SAVR 19 mm (Perimount Magna Ease) + CABG	Alive at 61 months
25	60/M	2013	21	43	Regurgitation	17		Severe	60	Leaflet tear	SAVR 23 mm (Perimount)	Alive at 48 months
26	62/M	2013	25	70	Regurgitation	24	3.3	Severe	60	Tear of the LCC	SAVR 25 mm (Perimount)	Alive at 20 months
27	73/F	2013	23	54	Stenosis	64	5.0	Mild	60	Leaflets calcification	TAVI 23 mm (S3)	Alive at 35 months
28	77/F	2013	21	1	Regurgitation	16	3.0	Severe	60	Tear of the NCC	SAVR 19 mm (Perimount Magna Ease)	Alive at 87 months
29	75/F	2013	21	49	Mixed	43	4.3	Moderate	68	Leaflets calcification	SAVR 21 mm (Perimount)	Alive at 39 months
30	73/F	2013	23	67	Regurgitation	14	2.4	Severe	40	Tear of the NCC	SAVR 21 mm (Perimount)	Alive at 21 months
31	79/F	2013	21	87	Mixed	71	5.3	Moderate	50	Leaflets calcification	TAVI 23 mm (S3)	Alive at 8 months
32	78/M	2013	23	72	Mixed	20	3.4	Severe	60	Tear of the NCC	SAVR 23 mm (Perimount)	Alive at 23 months
33	88/M	2013	23	74	Regurgitation	7	1.9	Severe	45	Leaflet tear	TAVI 23 mm (S3)	Alive at 16 months
34	80/F	2013	21	73	Mixed	80	5.3	Moderate	60	Leaflets calcification	SAVR 19 mm (Perimount) + CABG	Alive at 15 months
35	82/F	2014	25	57	Stenosis	71	5.2	None	60	Leaflets calcification	TAVI 26 mm (S3)	Alive at 23 months
36	77/M	2014	25	38	Regurgitation	15	2.8	Severe	52	Leaflet tear	TAVI 26 mm (S3)	Alive at 35 months
37	74/M	2013	21	67	Stenosis	84	5.3	Mild	69	Leaflets calcification	TAVI 23 mm (Evolute)	Alive at 28 months
38	80/M	2014	23	60	Mixed	22	3.1	Severe	60	Tear of the RCC	TAVI 23 mm (S3)	Alive at 18 months
39	63/M	2014	25	38	Regurgitation			Severe	60	Tear of the NCC	SAVR 23 mm (Perimount)	Alive at 39 months
40	53/M	2015	25	66	Regurgitation	15	2.7	Severe	55	Tear of the NCC	SAVR 23 mm (Perimount)	Alive at 2 months
41	80/F	2016	23	30	Regurgitation	8	2.1	Severe	60	Leaflet tear	TAVI 23 mm (S3)	Alive at 27 months
42	72/M	2016	25	49	Regurgitation		2.7	Severe	60	Leaflet tear	TAVI 26 mm (S3)	Alive at 9 months
43	82/F	2016	23	16	Regurgitation	8	2.1	Severe	60	Tear of the NCC	TAVI 23 mm (S3)	Died after 36 months
44	88/F	2018	23	31	Regurgitation		2.8	Severe	60	Leaflet tear	TAVI 23 mm (S3)	Died after 3 months

F: female; M: male; NCC: non coronary cusp; LCC: left coronary cusp; RCC: right coronary cusp; SAVR: surgical aortic valve replacement; TAVI: transcatheter aortic valve implantation.

**Figure 3:**
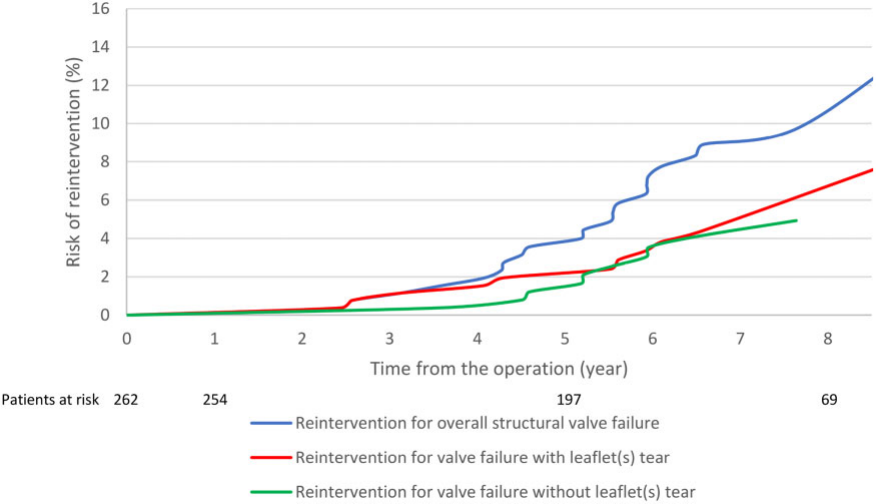
Cumulative risk probabilities of reintervention for overall structural valve failure, failure with and without leaflet(s) tear, using death as competing risk, for patients aged ≤70 years at the time of aortic valve replacement.

**Figure 4:**
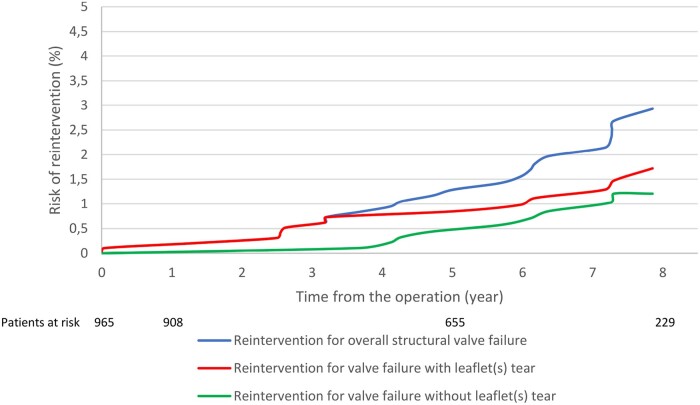
Cumulative risk probabilities of reintervention for overall structural valve failure, failure with and without leaflet(s) tear, using death as competing risk, for patients aged >70 years at the time of aortic valve replacement.

The cumulative incidence of reintervention for failure due to leaflet(s) tear was 0.16% (SE 0.11%), 1.08% (SE 0.29%) and 3.03% (SE 0.88%) at 1, 5 and 9 years, respectively. The cumulative incidence of reintervention for failure due to leaflet(s) calcification was 0, 0.7% (SE 0.24%) and 2.0% (SE 0.46%) at 1, 5 and 9-years, respectively. Figures 3 and 4 show the cumulative incidence of structural valve failure (overall, due to leaflets(s) tear and leaflet(s) calcification) using death as a competing risk for the overall population and for people aged >70 or ≤70 years at the time of Trifecta valve implantation. Supplementary Material, Fig. S1 reports the cumulative incidence of structural valve failure of Trifecta TF (5.2% at 9 years) and Trifecta TFGT (0.7% at 4 years).

## DISCUSSION

Several recent studies have reported a higher occurrence of early structural valve failure associated with Trifecta tissue valves when compared with other stented biological prostheses. A significant difference in terms of early durability was described in large cohorts of patients in terms of freedom from explant due to structural failure [2, 5, 6] or freedom from structural valve failure as detected by imaging assessment, intraoperative or autopsy findings [7, 12]. Our analysis was based on the hard end-point of reintervention (redo surgical aortic valve replacement or transcatheter valve-in-valve procedure) following degeneration of the implanted tissue valves. We found a cumulative incidence of reintervention due to Trifecta prosthesis degeneration of 5.11% at 9 years, which is in keeping with our previous study involving 836 patients operated on at Southampton General Hospital [5] and the results reported by Biancari *et al.* [6] and Yongue *et al.* [2] in populations with similar mean age at the time of valve implantation.

Modes of failure of Trifecta prostheses include leaflets calcification [2, 12], the development of fibrofatty circumferential pannus in the inflow portion [10] and, especially in the early period after valve implantation, leaflet(s) tear or dehiscence along the stent post(s) [5–7].

The occurrence of sudden leaflets tear due to mechanical stress has been occasionally reported in several case reports and small series [13–15] as further reviewed by Schaeffer *et al.* [16] and Kaneyuki *et al.* [8]. In a large series of 1058 patients followed at the University of Michigan, sudden onset of aortic regurgitation—mostly secondary to a partial cusp tear along a sent post—was found in 55% of the cases of Trifecta failure [7].

Similarly, in our study, we have found that leaflet(s) tear—due to non-infective disease—was the main mechanism of failure in 44 patients (≈55%) requiring reintervention after aortic valve replacement with a Trifecta prosthesis. Furthermore, we have highlighted that this mechanism was the leading cause of reintervention in the first 5 years and probably the reason accounting for the described higher occurrence of early structural failure of Trifecta valves when compared with other stented biological prostheses.

The design of Trifecta valves has been implicated as a potential cause of sudden cusp tear or dehiscence along with the stent post [2, 7, 12, 15]. The occurrence of leaflet(s) tear or dehiscence is a known mechanism of failure in externally mounted aortic valve prostheses. The first evidence was reported for the Hancock pericardial prosthesis and the Ionescu-Shiley valve [17]. The same mode of early failure (4.5–7 years after implantation) was subsequently described for the Mitroflow prostheses [18, 19]. The development of cusp(s) tear and dehiscence along the stent post was suggested to be independent of calcification and due to haemodynamic stress on the externally mounted leaflets [17, 18].

The results of a recent *in vitro* study conducted on Trifecta prostheses and using accelerated wear testing support the role of mechanical force in the development of tears at the level of valve commissures [20] and confirm the results of the ‘Dynamic failure mode’ test conducted on Trifecta valves within the structural performance testing before the premarket approval [21]. Five out of 9 Trifecta valves showed abrasion damage and at least 1 tear or hole around the stent post at 400 million cycles, corresponding to about 10 years of simulated cycling, and, in 2 cases, the occurrence of severe regurgitation at 600 million cycles, corresponding to about 15 years of simulated cycling. As stated by the authors [20], the *in vitro* setting did not account for the concomitant impact of biological components (i.e. blood rheological properties, immunological reaction); therefore, the observed durability during accelerated wear testing cannot be applied to *in vivo* scenarios as our analysis, in keeping with previous studies, found an earlier occurrence of failure by leaflet(s) tear. Nevertheless, this experimental assessment confirmed the growing clinical evidence of the early failure of Trifecta valves due to mechanical lesions.

On 6 July 2020, the UK Medicines and Healthcare Products Regulatory Agency issued a Medical Device Alert regarding early structural failure of Trifecta prostheses based on ‘65 UK adverse incident reports relating to 1st generation Trifecta and “improved” Trifecta valves; 5 relate to the Trifecta GT valve’. ‘The most common reported problems were leaflet damage and/or valvular insufficiency along with a range of other associated concerns. Time to failure, where known, ranged from perioperative to 8 years, with approximately half occurring between 2 to 3 years post implant’ [22]. The manufacturer acknowledged ‘that the design of the 1st generation Trifecta valve may increase the likelihood of early degeneration. Specifically, the SVD seen may be a result of having a valve design with externally mounted leaflets, in combination with a stent that may be deformed during implant’ [22].

UK Medicines and Healthcare Products Regulatory Agency advised clinicians to offer patients a ‘more frequent (enhanced) follow-up’ [22] that, despite the limitations caused by the COVID-19 pandemic, we have promptly established at University Hospital of Southampton and at the referral sites. Alongside the undoubtable advantage in improving patients’ safety, the wider availability of clinical and ultrasound imaging assessment will enable us to provide further and more precise evidence on Trifecta valves’ long-term durability and structural valve failure.

### Limitations

Our study has the limitations associated with a retrospective analysis. We acknowledge that reoperation represents a clinical decision and should not be used as a surrogate to estimate the occurrence of structural valve deterioration. Nevertheless, our primary aim was to delineate the modes of failure of Trifecta prostheses, and we were able to collect clear data from intraoperative and imaging assessments.

As pointed out by the manufacturer, there are some general precautions regarding proper valve sizing and handling that should be considered to minimize the risk of early prosthesis failure. We were not able to collect such technical information; however, we have already highlighted and discussed in our previous paper that the failure rate in Trifecta valves was similar among the surgeons operating at UHS and reflected the volume of valves implanted by each of them [5].

The later designs of the Trifecta valves are expected to reduce the risk of valve failure [22]. We found a low probability of early failure in the more recent Trifecta GT prostheses; however, we are able to present a limited follow-up and a smaller population for this subgroup; further data are needed to confirm a positive impact of these improvements.

## CONCLUSIONS

In a larger cohort of patients, we have confirmed our previous findings regarding the durability of Trifecta prostheses confirming a non-negligible risk of early failure. The occurrence of leaflet(s) tear is the main mechanism leading to an early reintervention up to 4–5 years since the implantation. Our experience showed that this mode of failure was often independent of leaflet(s) calcification. Trifecta GT valves exhibited a lower probability of early failure, but the population size and the available follow-up are still limited.

## SUPPLEMENTARY MATERIAL

Supplementary material is available at *ICVTS* online.

**Conflict of interest:** none declared.

## Data availability statement

The data underlying this article will be shared on reasonable request to the corresponding author.

## Author contributions

**Pietro Giorgio Malvindi:** Conceptualization; Data curation; Formal analysis; Investigation; Methodology; Writing—original draft. **Hassan Kattach:** Conceptualization; Data curation; Formal analysis; Supervision; Validation; Writing—review & editing. **Suvitesh Luthra:** Investigation; Methodology; Supervision; Writing—review & editing. **Sunil Ohri:** Conceptualization; Investigation; Methodology; Project administration; Resources; Validation; Writing—review & editing.

## Reviewer information

Interactive CardioVascular and Thoracic Surgery thanks Terezia B. Andrasi, Daniel-Sebastian Dohle, Leonard N. Girardi and the other, anonymous reviewer(s) for their contribution to the peer review process of this article.

## Supplementary Material

ivac086_Supplementary_Data

## ABBREVIATIONS

SAS: Statistical Analysis Software
SD: Standard deviation
