# Supplementary figures and images for: Modes of failure of Trifecta aortic valve prosthesis
Source: Interact Cardiovasc Thorac Surg. 2022 Mar 28;35(2):ivac086. doi: 10.1093/icvts/ivac086 (PMC9297516; doi:10.1093/icvts/ivac086)

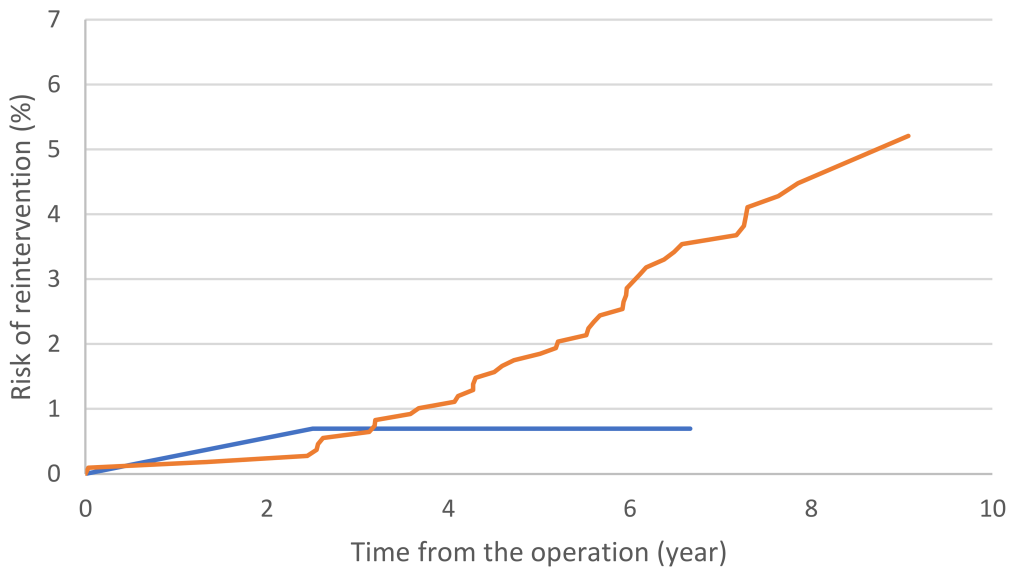

— Reintervention for valve failure TFGT — Reintervention for valve failure TF

Patients at risk 144 139  
1084 1023

74  
913

717

298

Supplement: ivac086_Supplementary_Data [file ivac086_supplementary_data.pdf]
